# Sensing of NO_2_, NH_3_, and C_3_H_6_O by graphene-Si Schottky diode at chosen voltage biases

**DOI:** 10.1038/s41598-025-94473-5

**Published:** 2025-03-21

**Authors:** Katarzyna Drozdowska, Janusz Smulko, Adil Rehman, Bartłomiej Stonio, Aleksandra Krajewska, Sergey Rumyantsev, Grzegorz Cywiński

**Affiliations:** 1 Department of Metrology and Optoelectronics, Faculty of Electronics, Telecommunications, and Informatics, Gdańsk University of Technology, G. Narutowicza 11/12, Gdańsk, 80-233 Poland; 2 Institute of High Pressure Physics PAS, CENTERA Laboratories, Warsaw, Poland; 3 Centre for Advanced Materials and Technologies CEZAMAT, Warsaw University of Technology, Poleczki 19, Warsaw, 02–822 Poland

**Keywords:** Electrical and electronic engineering, Graphene, Two-dimensional materials, Sensors and biosensors

## Abstract

Our research aims to propose a selective gas sensor based on a single graphene-silicon Schottky junction. By polarizing the sensor with selected voltage, we move between different regions of its gas sensitivity dependent on the lighting conditions. Our results show that low detection limits (36 ppb for NO_2_, 238 ppb for NH_3_, and 640 ppb for C_3_H_6_O) are obtained under UV irradiation at -0.4 V; however, a light source power supply is required. Then, the Schottky barrier height is primarily sensitive to gas adsorption. When the sensor operates in the dark, the characteristic region of graphene between the ohmic contact with the Ni/Au electrode and a Schottky contact with Si is responsible for observing the gating effect in the structure. It is visible as a bending in the *I-V* curve near 0.7 V, which shifts with the adsorption of gases due to potential induced by molecular dipoles. Observing and analyzing these two effects on DC characteristics makes our sensor a highly sensitive and selective platform of low-power consumption and low-cost methodology. Additionally, the applied Schottky junction between the graphene layer and the *n*-doped Si base makes the sensor more resistant to humidity adsorption during storage in ambient air than other graphene-based gas sensors (graphene back-gated FET).

**Supplementary Information:**

The online version contains supplementary material available at 10.1038/s41598-025-94473-5.

Graphene-based sensors and sensing systems have been of interest to scientists for at least a decade. The attractiveness of graphene is founded on its unique structural, optical, electrical, thermal, and mechanical properties^1,2^. Notably, the high activity of the two-dimensional (2D) surface, high carrier mobility, and unique transport properties gave rise to employing graphene as a highly sensitive probe in different types of sensors, including gas sensing devices^3,4^. Unfortunately, graphene employed as a resistive element in the gas detection systems was soon observed to be susceptible to low selectivity and fast aging when operating in ambient air with a certain percentage of relative humidity (RH). Additionally, the sensitivity of high-quality carbon nanomaterials is limited due to a low number of binding sites for analytes^5^. Therefore, various routes of enhancing the sensitivity, selectivity, and stability of the graphene-based sensors were tested, including decorating graphene surface with catalytic materials^6,7^, fabricating hybrid structures^8^, applying ultraviolet (UV) irradiation^9^, or employing different configurations of the devices, including field-effect transistors (FETs)^10^ and Schottky diodes^11^. The devices with the Schottky junction were observed to produce the same magnitude of sensing response to selected gases at a much lower voltage bias than FET, utilizing the same type of graphene grown on copper foil while also ensuring much lower susceptibility to the humid atmosphere^12^. When the 2D graphene layer is deposited directly on the semiconducting surface, such as a doped Si substrate, two materials form the Schottky junction (semimetallic graphene and semiconducting Si). The parameter that defines the homogeneity of such a junction is the ideality factor *η* (equal to 1 for an ideal diode)^13^. Graphene-based Schottky junctions usually exhibit ideality factors higher than 1, indicating that defects introduced during fabrication assist in the charge transport through the junctions^14,15^.

Initial studies on graphene-Si (G-Si) diodes in atmospheres of selected vapors showed that the adsorption of these vapors molecules modulates the characteristics of the junction. H. Kim et al. reported that the forward region of the *I-V* characteristic of the graphene-based diodes was mainly affected by nitrobenzene, chlorobenzene, benzene, and anisole^16^. The forward region of the characteristic at high voltage bias is dominated primarily by graphene resistance. Hence, changes in the conductivity profile of this 2D layer are mainly responsible for detection. The high positive voltage bias region moderately depends on the Schottky barrier high (SBH). However, at low forward bias, the *I-V* curve changes exponentially, so the series resistance of graphene does not contribute to the characteristic in this region. The reverse region of the *I-V* curve, which is susceptible to minimal changes in the Schottky barrier height (SBH), can also be affected by molecular adsorption, as demonstrated by M. Zhu et al.^17^. In this case, the prominent changes in the reverse current introduced by NO_2_, NO, and SO_2_ were associated with the decrease of SBH. Additionally, the investigation on G-Si diodes confirmed that different parts of the junction characteristics are influenced distinctly by different gases (NO_2_ and tetrahydrofuran under UV light)^12^. All these reports indicate that graphene-based Schottky junctions can serve as a sensing platform, and information about detected gases can be effectively derived from electrical measurements (forward/reverse currents at selected bias voltages). However, little has been reported about the mechanisms of detecting specific gases by G-Si Schottky diodes and the achievable limit of gas detection.

In this work, we investigated Schottky diodes fabricated with graphene and *n*-doped silicon to quantitatively detect selected gases (nitrogen dioxide, ammonia, acetone). We focused on studying *I-V* characteristics that can be divided into a few regions affected differently by selected target species and influenced by different mechanisms of gas detection. To enhance the sensitivity of the proposed sensor and reduce the detection limits, we applied UV irradiation and compared sensor responsivity at selected voltage biases. We observed that UV light shifts the sensitivity region of the G-Si diode to the reverse current regime. This reduces the power consumption of such a sensing device since, at -0.4 V voltage bias, currents flowing through the sensor are less than 100 nA under UV light activation. At the same time, the light-assisted operation of the sensor requires a UV LED power supply. Still, it can be significantly reduced when the sensor is nearer or even integrated with the UV light source switched on for a short period of gas sensing. On the other hand, we demonstrate that the gating effect introduced by dipoles of adsorbed molecules guides gas detection when no irradiation is employed. This effect occurs in the forward bias, around 0.7 V. Therefore, we show two different effects occurring in the same structure and propose the equivalent circuit for the G-Si device to analyze the adsorption/desorption processes related to the charge transfer and charge rearrangement at the molecule-graphene interface. This way, we can improve sensor performance by measuring DC characteristics only and adjusting the biasing conditions. Our demonstrated approach is more straightforward than specific fabrication routes, including doping the sensing material to increase its sensitivity and selectivity.

## Methods

### Graphene-silicon diodes fabrication

**Fig. 1:**
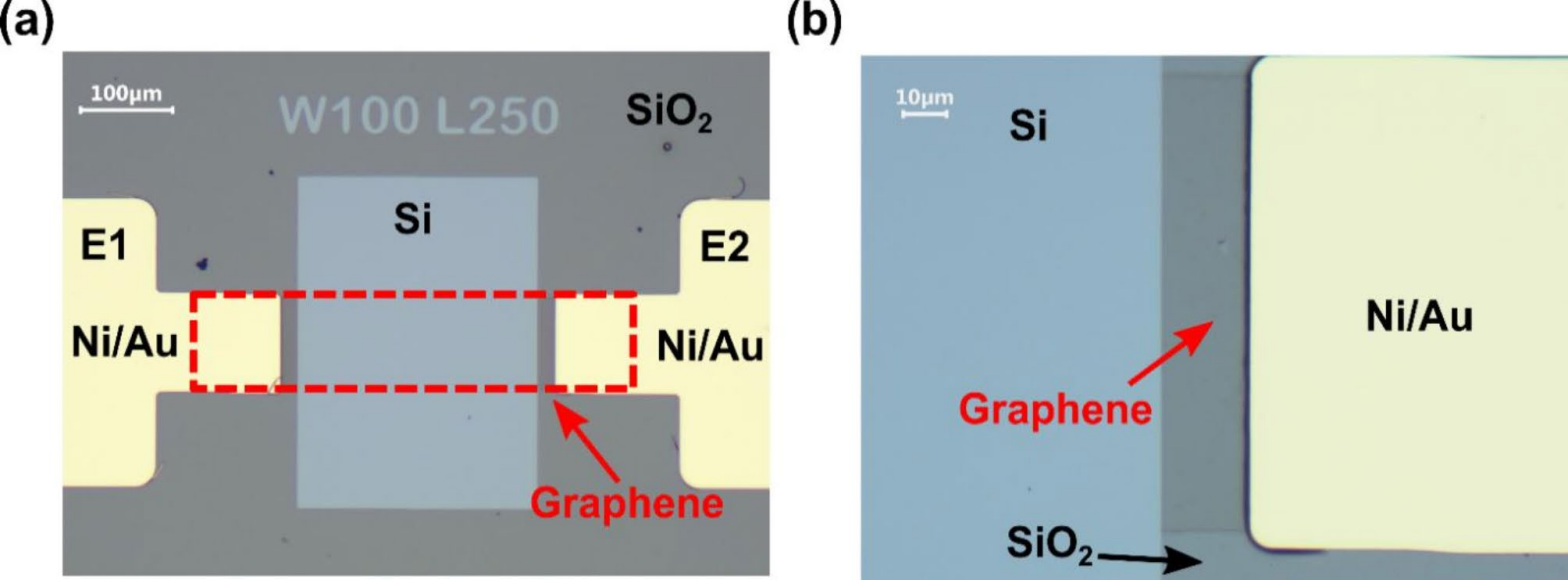
Optical microscopic images in **(a)** 100x and **(b)** 500x magnification of the G-Si Schottky diode with junction area of 25·10^3^ μm^2^ (width = 100 μm, length = 250 μm). In **(a)**, the grey area in the middle is the silicon well, and the region marked as a red dashed rectangle shows the deposition placement of graphene. In higher magnification, the graphene layer is visible as a shadowed region between the Si area and Ni/Au contact. SiO_2_ is the grey background surrounding the Ni/Au contacts (E1 and E2) and Si part, and it lies beneath the graphene layer in some parts.

We started fabrication of the Schottky diodes by cleaning the *n*-type silicon wafer and growing 90 nm of the thermal oxide (SiO_2_) on its surface. Ellipsometry measurements confirmed the final thickness of the SiO_2_. We used commercial Si of 1–10 Ω·cm resistivity and carrier concentration between 5·10^14^ and 5·10^15^. Next, the windows in SiO_2_ were selectively etched for the following graphene transfer. CVD-graphene grown on Cu-foil (from Graphenea) was transferred to the Si/SiO_2_ substrate by electrochemical delamination as described elsewhere^18^. Afterward, reactive-ion etching (RIE) in oxygen plasma was applied to selectively etch graphene and create such a pattern that part of the graphene layer lies on Si and part is on SiO_2_. Ni/Au (10/200 nm) contacts were then deposited using a thermal evaporator on the graphene lying on SiO_2_. Figure 1 shows the optical microscope image of one of the devices. All the lithography processes were performed by photolithography. Transfer of graphene from Cu foil supported with PMMA enables the deposition of large areas of graphene (up to a few cm in size) of reproducible structural features for samples produced within the same batch. The variance between the consecutive batches obtained from different commercial graphene/Cu foils was not observed in the graphene structure but rather in electrical properties after the deposition of contacts. This is the main limitation of the proposed transfer procedure, however, sensor working conditions can be calibrated after DC characteristics collection so that the sensors operate in the same power consumption region. A more detailed description of the graphene-silicon diodes fabrication can be found in our previous work^12^. For gas sensing experiments realized in this work, we selected graphene-silicon diodes with an active area of 25·10^3^ μm^2^ (width = 100 μm, length = 250 μm) and 5·10^4^ μm^2^ (width = 200 μm, length = 250 μm).

### DC characteristics measurements

DC response measurements were performed in an MPS150 probe station from FormFactor. Keithley-4200 A-SCS parameter analyzer with two medium power source-measure units (type 4201-SMU) was used for all electrical measurements. Each characteristic was measured from 0 to + 2 V or from 0 to − 2 V and combined in one graph. It reduced the detrimental effect of discharging deep areas of *n*-doped Si during the measurements, inducing an eventual shift of the recorded *I-V* characteristics. Some of the measured devices exhibited a shift of *I-V* characteristic (no more than 0.3 V) due to unavoidable differences in temperature between the studied structure and the needle probes, inducing a thermoelectric voltage in the input measurement circuit (see supplementary Figure S1 showing an exemplary *I-V* characteristic with the shift). One contact was connected to the graphene (through Ni/Au contact), while another was connected to the silicon (etched and metalized backside of the Si wafer). Negative voltages represent reverse bias, and positive voltages represent forward bias for the graphene/*n*-Si junction. During all measurements, the probe station with the sensor was kept inside a nontransparent box to limit interferences from ambient light and laboratory airflow. The metal gas chamber of ~ 120 cm^3^ volume with gas inlet and outlet was employed to maintain the chosen gas atmosphere surrounding the sensor. Light-assisted sensing was implemented using a UV LED (ProLight Opto, type PB2D-1CLA-TC, *λ* = 275 nm, with an optical power density of 1.59 mW/cm^2^). The LED was positioned approximately 0.5 cm from the sensor surface in the gas chamber. UV light shifts slightly the point of minimum current in the *I-V* characteristic. The observed shift was around tens of mV at the maximum when compared with the *I-V* characteristic recorded in the dark, which mainly resulted from the photo-voltage effect. The repeatability of the electrical properties of the junction was monitored each day by measuring the *I-V* curve in laboratory air in the dark before initializing sensing experiments to control any possible aging of the sensor and drift in the baseline parameters.

### Gas-sensing experiments

Nitrogen dioxide (NO_2_), ammonia (NH_3_), acetone (C_3_H_6_O), and chloroform (CHCl_3_) were used as target gases for gas sensing experiments with the G-Si diodes. Dry synthetic air (S.A.) was used as a carrier gas and a reference atmosphere for the sensor response studies. We mixed S.A. with target gases at specific proportions to obtain selected concentrations: 0.05–7 ppm of NO_2_, 1–15 ppm of NH_3_, and 10–30 ppm of C_3_H_6_O. To produce chloroform vapor, S.A. was transferred through the glass bubbler with the liquid solvent. This way, the output concentration of vapor is controlled with the carrier gas flow. 100 ppm of CHCl_3_ concentration was obtained by transferring 50 mL/min of S.A. through the liquid. Experimental detection limits were the lowest investigated concentration values for each gas, for which we observed the measurable response. During the admission of the gases to the sensing chamber, we maintained a constant overall gas flow of 50 mL/min regulated by mass flow controllers (Analyt-MTC, model GFC17). Before starting sensing experiments with target gases, we admitted the carrier gas (in the dark or under UV irradiation) for ~ 1 h to stabilize the baseline properties of the sensor and reduce drift. To produce the humid carrier gas, we transferred dry S.A. through the glass bubbler with deionized water. Since the overall gas flow produces water vapor of specific relative humidity (RH), we obtained RH of ~ 40% by keeping the constant gas flow of 50 mL/min. All sensing experiments were conducted at room temperature (RT ~ 25 °C) and ambient pressure (~ 1 bar). The sensing responses of the G-Si diode were presented as the relative change in the current flowing through the sensor *I*_S_ in the presence of selected gas and the current *I*_0_ measured in the reference atmosphere (S.A.) at specific voltage bias: (*I*_S_-*I*_0_)/*I*_0_.

### Detection limit (DL) Estimation

Theoretical detection limits (DLs) were estimated based on the dependence between (*I*_S_-*I*_0_)/*I*_0_ values derived from the *I-V* characteristics and selected concentrations of ambient gases. Current values at voltage bias *V* = 0.7 V and *V* = -0.4 V were chosen for the dark and under UV light measurements, respectively. The fourth-order polynomial function was fitted to the experimental results, and the root mean square was calculated from the difference between the polynomial fit function and experimental points. Next, a linear fit was performed in the quasi-linear region of the (*I*_S_-*I*_0_)/*I*_0_ vs. concentration dependence. The quasi-linear region of sensing responses refers to the experimental points that follow linear-like dependence without saturation of the response. DL was then calculated based on the formula: DL = (S/N·RMS)/slope, where S/N is the signal-to-noise ratio equal to 3, RMS is the root mean square derived from the difference in the polynomial fit and experimental points, and the slope (equal to sensor sensitivity in linear response range in 1/ppm units) is derived from the linear fitting. The same procedure of DL estimation can be found elsewhere^19^.

### Results and discussion

We investigated exemplary Schottky diodes with graphene and *n*-doped Si substrate for experiments with selected gases. A Schottky junction is created after contacting graphene of metal-like conductivity and semiconducting *n*-doped Si. Only selected substrate parts were etched to create Si wells (light grey rectangle in Fig. 1), and the rest were covered with SiO_2_. Graphene was transferred on the Si well, and metal Ni/Au contact pads were deposited on the parts where graphene lies on SiO_2_ (a shadowed region visible in Fig. 1b). The area of the investigated junction is 25·10^3^ μm^2^ (width *W* = 100 μm and length *L* = 250 μm as depicted in Fig. 1a). We used monolayered graphene grown by chemical vapor deposition (CVD) on Cu foil, characterized by a high density of surface defects after post-growth processing and transfer to Si/SiO_2_ substrates. The edges and point defects in graphene structure are beneficial for gas adsorption, as reported elsewhere^20,21^. Additionally, the aging of graphene may result in differences at the G-Si interface and create a characteristic bending in the DC characteristic of the G-Si diode (a gating effect). More details on the morphology of graphene used to produce Schottky diodes can be found elsewhere^22^. Moreover, such configuration of the graphene-based device prevents graphene from fast aging (induced by water molecules adsorbed from laboratory air) compared to FETs with graphene channel, as demonstrated before^12^, and is a great advantage for any practical application of such sensors.

**Fig. 2:**
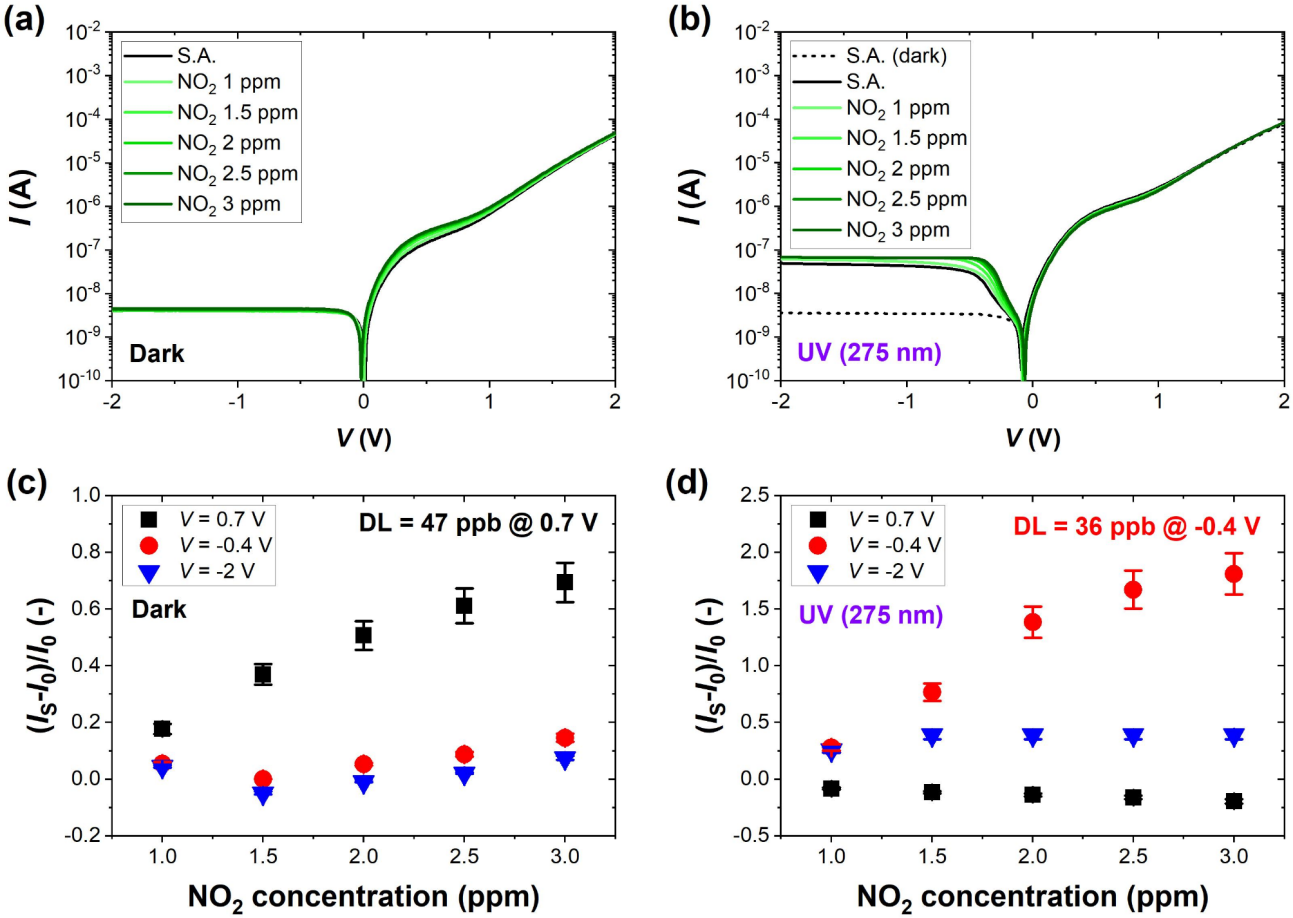
Current-voltage characteristics of the investigated G-Si Schottky diode measured in S.A. and selected concentrations of NO_2_ (1–3 ppm) **(a)** in the dark and **(b)** under UV light (275 nm, 1.59 mW/cm^2^) and the sensor response presented as the relative change in the current flowing through the sensor *I*_S_ at selected voltage bias for **(c)** dark and **(d)** UV light conditions. The black dashed curve in **(b)** is the result for dark conditions to show the effect of UV light in the reverse regime. DL marked on the graphs was estimated for the voltage bias at which the responses were the highest (0.7 V in the dark and − 0.4 V under UV light). The error bars represent the accuracy of current measurements.

We selected three gases for evaluation of the gas-sensing performance of the G-Si diode: inorganic and strongly oxidizing nitrogen dioxide (NO_2_), inorganic and reducing ammonia (NH_3_), and organic acetone (C_3_H_6_O) or chloroform (CHCl_3_). They belong to volatile organic compounds (VOCs) that usually exhibit reducing properties when adsorbed on different materials^23^. However, their adsorption energies are relatively low, compared explicitly to NO_2_ and other inorganic species and the carrier exchange between molecules and sensing material is poor, so their effective detection in ambient conditions with sub-ppm limits remains challenging^24^. NO_2_ is a highly toxic pollutant produced in large-scale industrial processes and car exhaustion, so its detection is an essential practical problem. The *I-V* characteristics obtained for 1–3 ppm concentration of NO_2_ are depicted in Fig. 2. In the dark, the forward region of the characteristic is mostly active toward varied concentrations of NO_2_. The most visible differences induced by the target gas are around a voltage bias of 0.7 V, where the characteristic bends (see Figure S2a for magnified region of high gas responsivity in linear scale). At the same time, for higher positive voltage as well as in the reverse region of the characteristic, the current responses are minor in the dark. In comparison, under UV light, the active region of the sensor is shifted to the reverse current regime (below 0 V). Particularly, the region near the bent part of the curve around − 0.4 V is the most sensitive to NO_2_ (see Figure S2b for magnified region in linear scale). For negative voltages near − 0.4 V, the changes in the current are also visible and are more pronounced than in the forward region near 0.7 V. The reverse region is susceptible to changes in the SBH, which, in this case, is vividly affected by the NO_2_ adsorption. Based on our results, we could split the G-Si diode *I-V* characteristic into the following regions, that is, voltages below 0 V (the minimum current point in log scale), the region of positive voltage bias between 0 V and where the bending ends from to ~ 1.2 V, and higher positive voltages over 1.2 V. Depending on the lighting conditions, we move between the regions of high gas sensitivity utilizing the same device. Based on the results obtained for selected concentrations of NO_2_, we estimated the detection limit (DL) for the optimum voltage biases. In the dark, DL reached 47 ppb at 0.7 V, and it was reduced under UV light to 36 ppb at -0.4 V, as presented in Fig. 2c and d. The current response reached ~ 69% to 3 ppm of NO_2_ in the dark under optimum voltage bias and ~ 181% to the same concentration under UV light. It signifies that UV irradiation enhances the sensitivity of the G- Si sensor toward NO_2_ when the optimum voltage bias is used. The measurement for NO_2_ under UV light was repeated for another sample with a G-Si junction of the same dimensions to check the reproducibility of the sensors responsivity produced within the same batch. The results demonstrated in Figure S3 show that the effect of gas adsorption is visible mainly in the reverse regime, and the direction of changes is maintained. We also want to highlight that before introducing such sensors to practice, more experiments on reproducibility within the same and different production batches should be performed.

**Fig. 3:**
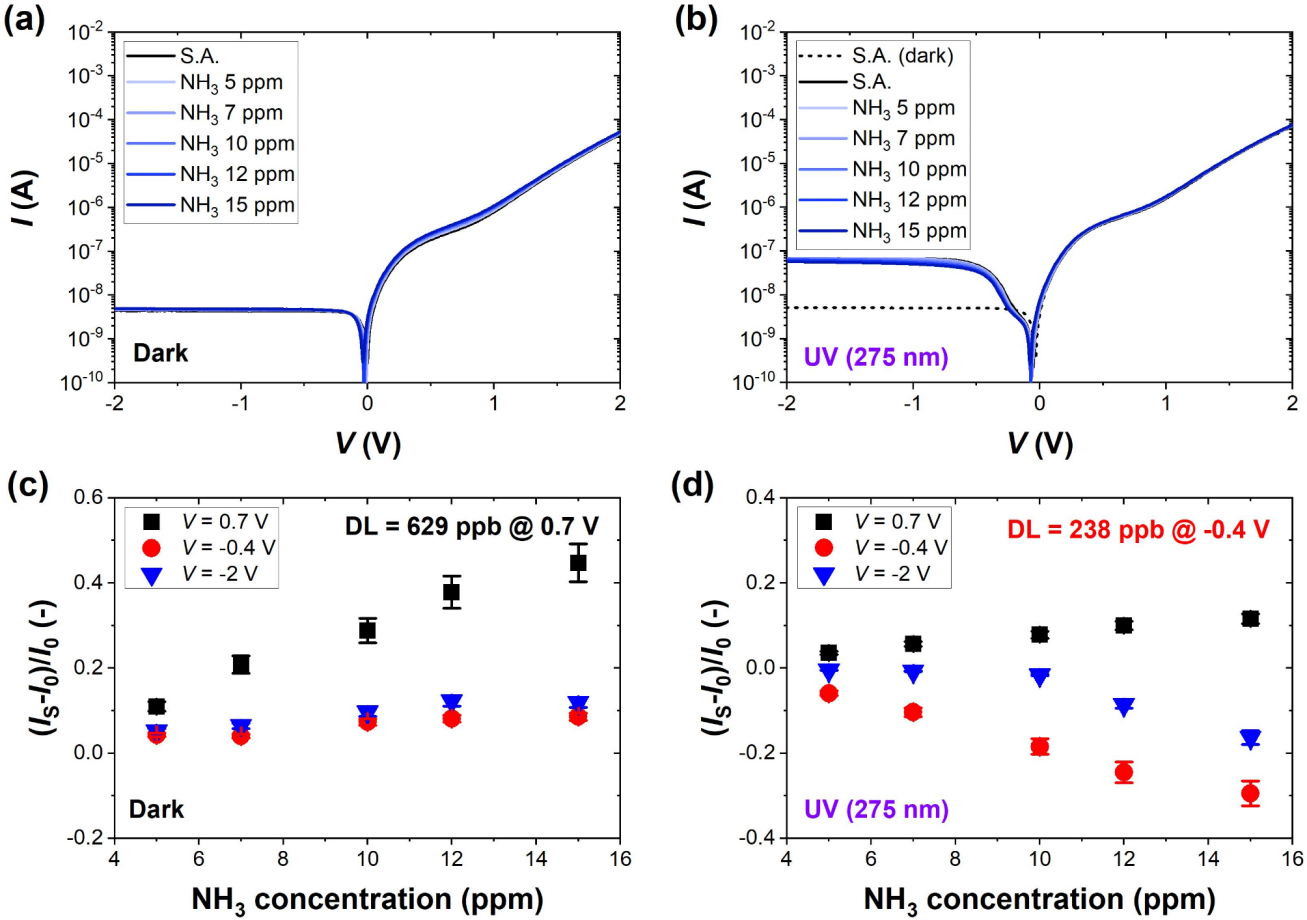
Current-voltage characteristics of the investigated G-Si Schottky diode measured in S.A. and selected concentrations of NH_3_ (5–15 ppm) **(a)** in the dark and **(b)** under UV light (275 nm, 1.59 mW/cm^2^); and the sensor response presented as the relative change in the current flowing through the sensor *I*_S_ at selected voltage bias for **(c)** dark and **(d)** UV light conditions. The black dashed curve in **(b)** is the result for dark conditions to show the effect of UV light in the reverse regime. DL marked on the graphs was estimated for the voltage bias at which the responses were the highest (0.7 V in the dark and − 0.4 V under UV light). The error bars represent the accuracy of current measurements.

Similar measurements for 5–15 ppm of NH_3_ revealed the repeatable pattern observed on the *I-V* characteristics (see supplementary Figure S4 for characteristics with selected magnified regions of *I-V* curves where responsivity to NH_3_ is the highest). Again, the sensor responded to increasing concentration of the target gas in the forward region (bending region) in the dark (Fig. 3a), but under UV light, the current responses were the highest in the reverse region (Fig. 3b). Notably, the responses to ammonia are quantitatively lower than to NO_2_. Our observation is consistent with the results obtained from first-principle studies that show the weaker interaction between ammonia and graphene due to the lower adsorption energies of this molecule^25,26^. DL estimated for NH_3_ is 629 ppb in the dark at 0.7 V. Still, it is more than 2.6 times decreased with the aid of UV irradiation to 238 ppb at -0.4 V. Interestingly, NH_3_ produces sensing responses of the same sign as NO_2_ in the dark at 0.7 V (Figs. 2c and 3c) and of the opposite sign under UV light at -0.4 V (Figs. 2d and 3d). The observation of current changes under UV light is more straightforward and expected due to the opposite direction of charge transfer between the molecules of electron-accepting NO_2_ or electron-donating NH_3_ and graphene. At the same time, it signals that different mechanisms guide the detection process in the dark and under UV light, and we can observe both these effects solely with *I-V* characteristics.

**Fig. 4:**
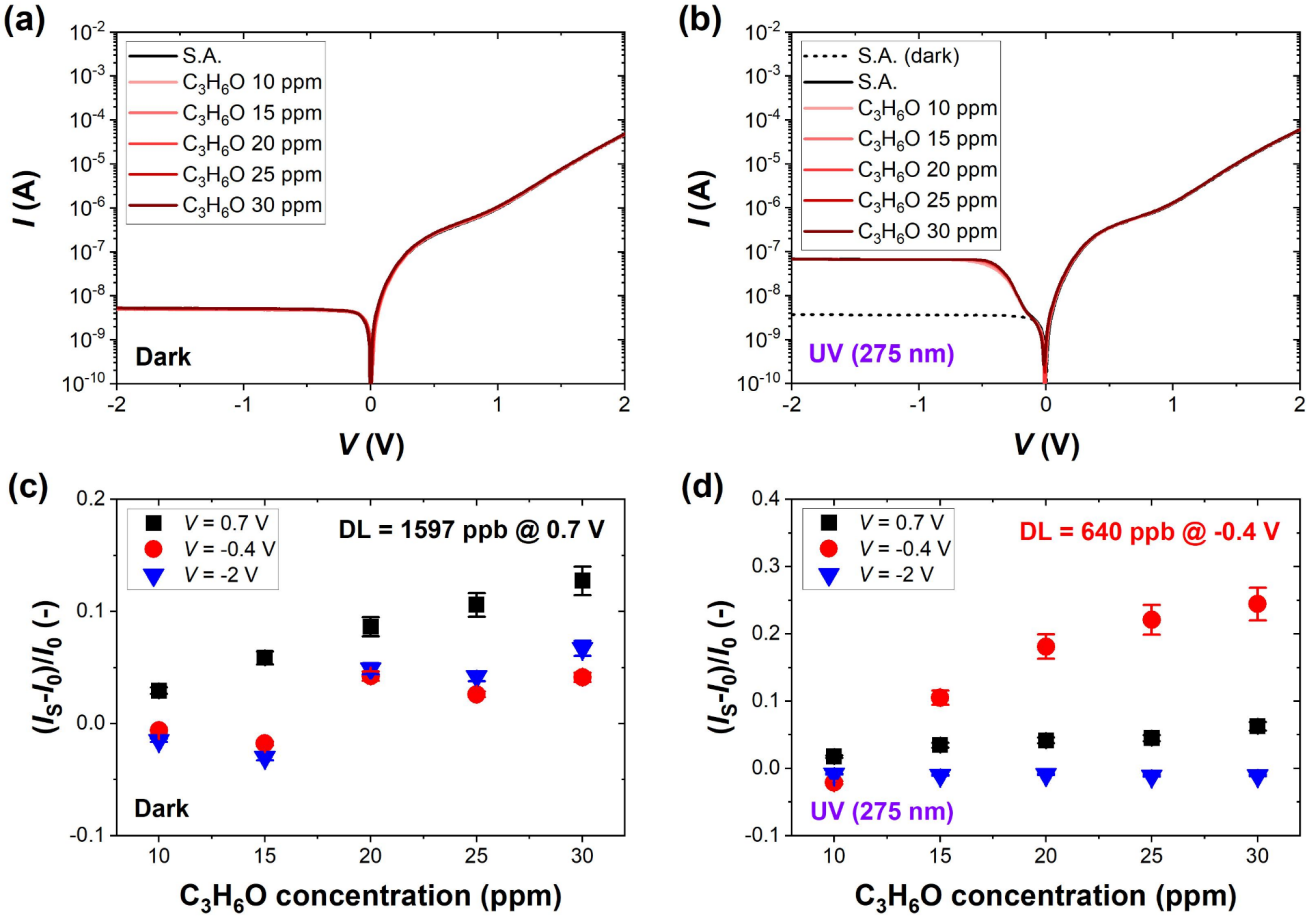
Current-voltage characteristics of the investigated G-Si Schottky diode measured in S.A. and selected concentrations of C_3_H_6_O (10–30 ppm) **(a)** in the dark and **(b)** under UV light (275 nm, 1.59 mW/cm^2^); and the sensor response presented as the relative change in the current flowing through the sensor *I*_S_ at selected voltage bias for **(c)** dark and **(d)** UV light conditions. The black dashed curve in **(b)** is the result for dark conditions to show the effect of UV light in the reverse regime. DL marked on the graphs was estimated for the voltage bias at which the responses were the highest (0.7 V in the dark and − 0.4 V under UV light). The error bars represent the accuracy of current measurements.

The measurements conducted for acetone (Fig. 4) revealed that the G-Si sensor responds to this gas in a less obvious manner (see supplementary Figure S5 for characteristics with selected magnified regions of *I-V* curves). In the dark, there are only minor variations in the current induced by adsorption of 10–30 ppm of the organic gas (Fig. 4a), and the proportionality between the current response and gas concentration is visible only for the forward voltage bias (0.7 V). For such voltage polarizing the active area of the sensor, the detection limit significantly goes beyond 1 ppm. Under UV light, the most reliable response is obtained in the reverse region of the *I-V* curve with characteristic bending (Fig. 4b). The current responses reach 24% for 30 ppm of acetone at -0.4 V, and the theoretical DL estimated at -0.4 V is reduced 2.5 times (to 640 ppb) compared to dark conditions (see Fig. 4c and d). However, our results suggest that experimental DL for acetone is close to 10 ppm; as for such concentration, the responses were only between 1 and 2% under UV light. Moreover, we ran additional experiments with lower concentrations of NO_2_ and NH_3_ to establish the experimental DL for these gases. Figure S6 shows the *I-V* characteristics measured for NO_2_ (50–1000 ppb) and NH_3_ (1–7 ppm). The obtained results suggest that NO_2_ concentration between 50 ppb and 100 ppb is a practical detection limit for this gas (response of a maximum of a few % obtained for selected voltage biases). In the case of NH_3_, the responses for concentrations below 3 ppm are significantly under 10% (at *V* = 0.7 V or *V* = -2 V) or do not even correlate directly with gas concentration (at *V* = -0.4 V), signaling experimental DL in the ppm range. Experimental DLs are higher than theoretical ones, particularly for ammonia and acetone, but it is important to note that experimental DL depends on the used setup and measuring units. We underline that additional measurements were done after a few months delay. It can have an impact on the measured DL values. Thus, quite similar results for so low gas concentrations confirm reasonable time stability of the studied sensors. Still, the G-Si Schottky diode sensor explicitly responds to inorganic species, whereas its sensitivity to organic acetone is lower, possibly due to weaker interaction and exchange of carriers between organic molecules and graphene surface. Thus, in the following measurements, we studied the time responses of the G-Si sensor to NO_2_ and NH_3_ only.

**Fig. 5:**
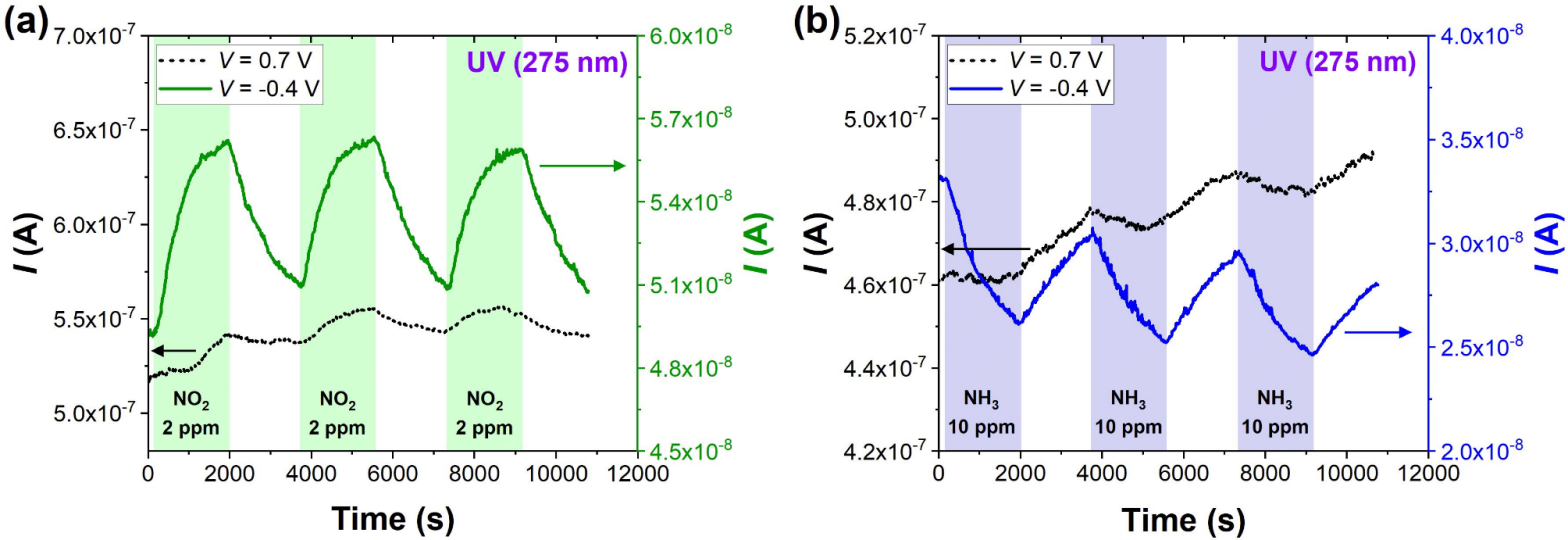
Time response (current vs. time) of the G-Si Schottky diode to the repeatable cycles of introducing **(a)** NO_2_ (2 ppm) or **(b)** NH_3_ (10 ppm) under UV light (275 nm, 1.59 mW/cm^2^) at selected voltage bias. The left axes (black) correspond to the measurements at *V* = 0.7 V, and the right axes (green/blue) designate currents measured at *V* = -0.4 V.

Figure 5 compares the time response of the G-Si Schottky diode toward repeatable cycles of introducing 2 ppm of NO_2_ and 5 ppm of NH_3_. Time-domain measurements were limited to the UV light case only, as the initial investigation proved that light assistance enhances the sensitivity and improves DLs of the G-Si sensor. For comparison, time responses were collected at two voltage biases, 0.7 V representing the forward bias bending of the characteristic and − 0.4 V representing the reverse bias bent part of the *I-V* curve. Notably, UV irradiation induces higher and more stable changes in the current flowing through the sensor at -0.4 V. Even though the currents measured at -0.4 V are one order magnitude lower (tens of nA) than at 0.7 V (hundreds of nA), the sensing responses are more stable, and the drift of the baseline is less pronounced. Due to the drifting baseline at 0.7 V, the time response to NH_3_ is less apparent – response/recovery cycles show that NH_3_ decreases the current, but the overall current change is positive during the whole measurement. Due to that, the reverse voltage bias ensures higher sensitivity and stability of time-domain measurements. Time-resolved studies also confirm that the magnitude of responses to NO_2_ and NH_3_ are repeatable in consecutive cycles of adsorption/desorption, which is a crucial parameter for prototype sensors and indicates sensor stability in short and long time^27,28^. Moreover, we ran additional experiments with humid air (RH of ~ 40%) as carrier gas (see Figure S7). The results show that water molecules significantly limit the sensitivity to NO_2_ and NH_3_ even under UV light. Particularly in the case of NO_2_ (1–7 ppm) and lower concentrations of NH_3_ (5–10 ppm), the physisorption of H_2_O competes with the adsorption of target gases on graphene binding sites. Thus, the effect of the humid atmosphere must be considered for realizing detection systems based on G-Si Schottky diodes for real-world applications, and a broader investigation is required. We underline that this effect is much lower when compared with other graphene-based gas sensors (e.g., graphene back-gated FETs)^22^.

Simple measurements of the DC characteristics of the G-Si device revealed interesting phenomena induced by lighting conditions and ambient gases. By dividing the *I*-*V* curves into several regions of varied gas sensitivity, one can study different mechanisms of gas adsorption using a single sensing device. We highlight two principal effects observed from the measured DC characteristics; the first one occurring in the reverse current regime is associated with the changes in the SBH, and the second observed in the forward current region at the characteristic bending. These two effects drive gas detection and affect the point of maximum sensor sensitivity depending on the used UV light assistance.

**Fig. 6:**
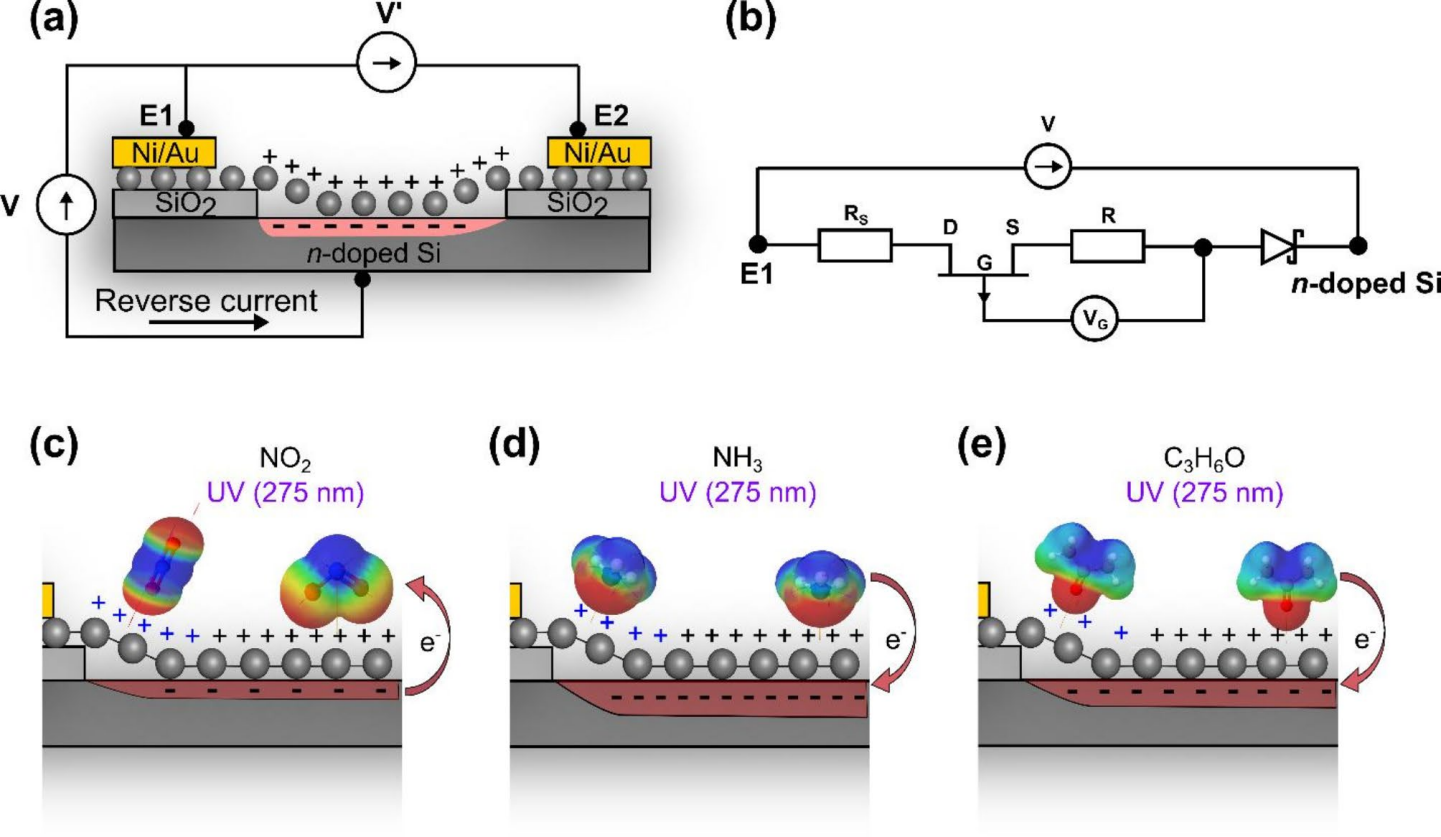
**(a)** Schematic representation of the G-Si Schottky diode sensor in the reverse voltage bias – graphene behaves as a *p*-type semiconductor in air; thus, there is an accumulation layer of electrons at the G-Si interface and depletion of electrons at the surface of graphene (representing the Schottky barrier). The equivalent circuit in **(b)** proposed for the G-Si sensor consists of the sensor resistance *R*_S_ dominated by resistance of graphene and Ni/Au contact electrode, field-effect transistor part with voltage bias *V*_G_ induced by dielectric dipoles of the adsorbed gas molecules, and the Schottky diode representing the junction between graphene and Si. The process of molecular adsorption under UV light in the reverse current regime is depicted for **(c)** NO_2_, **(d)** NH_3_, and **(e)** C_3_H_6_O. Additional voltage *V*’ can be used to measure characteristics of the graphene layer as a function of *V* across the Schottky junction.

In the dark, the sensor maximum responsivity to target gases is visible around 0.7 V, where the characteristic bends. This distinct bending can be associated with the part of the sensor between the Ni/Au electrode and Si substrate, where deposited graphene creates a sort of transitional region between lying on the oxide layer and forming a junction with Si. It can be visualized as an incline in the graphene layer, as depicted in the schematic cross-section of the studied device in Fig. 6a. This region plays an important role when the current flows through the sensor, particularly under the forward voltage bias, as this is the shortest path for electron transfer between the Ni/Au electrode and the Si substrate. This part of the structure can be susceptible to any potential induced near the graphene surface, which may create a gating effect similar to the one observed in the graphene-based FETs. However, the gating effect can be present for the whole graphene layer that is isolated from Si by the energy barrier, which results in graphene properties dependent on the applied voltage. During gas detection by the G-Si device, the graphene structure is affected by dipoles of the adsorbed gas molecules that increase the gating potential. The larger the number of adsorbed dipole molecules, the higher the potential induced. Thus, the forward region of the *I*-*V* characteristic shifts with increasing concentration of target gases. Interestingly, the direction of changes in the sensor characteristic is the same, independently of the gas type, and only the magnitude of the responses can be used to determine the type of gas molecules and their concentration. We depicted this part of the G-Si sensor as the *p*-type channel FET with gating voltage induced in the equivalent circuit in Fig. 6b, which the adsorption of different gases can modulate. This model was confirmed by considering additional DC voltage source *V*’ between the Ni/Au electrodes E1 and E2 (Fig. 1) and measuring DC characteristics of the sensor current *I* versus *V*’ in function of voltage *V* (Figure S8a). The results (Figure S8b) confirmed our assumption that the voltage *V*’ impacts *V*_G_ and the recorded characteristics *I*(*V*,*V*’) correspond to the characteristics of the FET transistor in linear region to some extent. Another interesting observation is that under UV light, this part of the device becomes less sensitive, which suggests that part of the measurements for gas detection can be conducted in the dark, which reduces the energy consumption associated with UV LED power supply and limits the power required for sensor operation only to produce 0.7 V of voltage across the sensor (less than a microwatt). Thus, the most significant advantage of using the forward bias measurement is the possibility of using the device in the dark without an additional power supply to irradiate the sensing surface.

As mentioned above, the peak of sensor sensitivity shifts toward the reverse current regime under UV light. The irradiation induces the surface cleaning effect by desorbing any eventual humidity molecules and part of the oxygenic species and generates weakly bounded oxygen photo-ions, encouraging the surface binding sites for target gas molecular adsorption. Notably, the exponential part of the DC characteristic at ~-0.4 V was observed to respond the most to the selected concentrations of target gases. During irradiation, around ten times higher reverse current flows through the sensing device. The Schottky barrier at the G-Si interface (as illustrated in Fig. 6a) is then primarily sensitive to molecular adsorption, and charge transfer between molecules and graphene drives the gas detection. Since NO_2_ is an electron-accepting molecule and readily forms NO_2_^-^ ions by withdrawing electrons from the sensing layer, the SBH decreases. For electron-donating NH_3_, the effect is the opposite, and SBH increases moderately, whereas for acetone, the effect on the SBH is the weakest. The schematic illustration of how the energy barrier at the G-Si interface changes is shown in Fig. 6c (NO_2_), Fig. 6d (NH_3_), and Fig. 6e (C_3_H_6_O). The change in SBH is directly correlated with the electron-accepting or -donating properties of the analytes; thus, the shifts in the DC characteristics are in opposite directions for oxidizing and reducing gases, which facilitates distinguishing between species. By selecting operating conditions (voltage bias), we can enhance the selectivity of the sensor. The selectivity factor can be estimated by comparing the sensitivity of two different gases at the same operating conditions. In the case of our sensor, the current response at *V* = -0.4 V under UV light is 1.81 for NO_2_, -0.29 for NH_3,_ and 0.24 for acetone, meaning that the response to NO_2_ is 6.2 and 7.5 times higher (the absolute values) than from other reducing gases (for the highest considered concentration for each gas). A similar estimation for dark conditions and *V* = 0.7 V reveals that the response to NO_2_ is 1.5 and 5.3 times higher than for NH_3_ and C_3_H_6_O, respectively. Therefore, the selectivity factor (the quotient of current responses) is lower in the dark. To further support our view that the investigated sensor exhibits explicit sensitivity to NO_2_ at selected voltage bias and under UV light, we performed measurements for chloroform (CHCl_3_) of 100 ppm concentration that induced the same direction of changes in the *I-V* curve as NO_2_. It can be seen from Figure S9 that two orders of magnitude higher concentration of CHCl_3_ compared to NO_2_ induces 14-times lower current response (at *V* = -0.4 V, the response to 100 ppm of CHCl_3_ is 2%, and to 1 ppm of NO_2_ is 28%). This supports our statement on increased selectivity to NO_2_ under particular biasing and lighting conditions. Furthermore, the shape of the bent part of the characteristic changes differently with investigated gases, which can additionally provide valuable information for gas sensing when applying more advanced detection algorithms. A lower voltage is needed to induce the optimum sensitivity with the UV-irradiated sensor; however, an additional LED power supply is necessary. Overall, the power consumption for the sensor operating efficiently in the reverse regime of its characteristic is ~ 500 mW altogether but can be reduced an order or so when the UV LED is integrated with the sensor and placed at a very short distance. The power required for polarizing the G-Si sensor is up to a few orders of magnitude lower compared to other reported sensing devices based on graphene, such as chemiresistors, FETs, or other Schottky junctions that often require power in the mW range to operate effectively in the dark^22,29^.

## Conclusions

Our work demonstrates a graphene-silicon Schottky junction that can be a sensitive and selective gas sensor when the information about the sensing responses is derived from *I*-*V* characteristics in the dark and under UV irradiation at particular voltage biases. Reduced detection limits were obtained under UV light under reverse voltage bias for all investigated gases. The same part of the DC characteristic was sensitive to electron-accepting or -donating properties of the target species, which helps determine the oxidizing or reducing properties of the detected gas and easier discrimination of analytes. However, the forward voltage region became more active toward gases without any light assistance, which is ascribed to inducing gating potential near the graphene during dipole interaction with the sensing surface. The simple measurement of the *I*-*V* characteristic of a single G-Si Schottky diode is a base for analyzing gas sensing responses. Utilizing the information from the measurement conducted in the dark under forward bias (*V* = 0.7 V) and under UV light under reverse bias (*V* = -0.4 V), we are able to distinguish between the studied target species (NO_2_, NH_3_, C_3_H_6_O). Therefore, we demonstrate that a single device based on the G-Si Schottky junction can be an efficient sensing platform for simplified discrimination of target gases with only one device but using its different parts, contributing to the overall DC characteristic. The possibility of conducting part of the sensing measurements in the dark under forward voltage bias reduces the overall power consumption; however, it is at the cost of lower sensitivity and selectivity compared to the reverse regime, where changes in SBH guide the gas detection. Moreover, enhancing the sensitivity and selectivity of the proposed devices by the method of measurements limited only to collecting the response data with *I*-*V* characteristics points toward a simple and low-cost gas detection system. The method is also beneficial against more complicated fabrication procedures, including doping the gas-sensitive material to obtain high sensitivity and selectivity. Additionally, the sensor does not require intermittent cleaning between consecutive days or even weeks of measurements at least, which is a great advantage compared to graphene-based FETs^22^. Repeatable characteristics for investigated G-Si diodes were observed for around three months of consecutive measurements, suggesting the stability of the proposed devices in a longer term and at least partial resilience of baseline electrical parameters toward humidity in ambient air. The effect of sensor storage in humid air (standard RH in laboratory conditions of 30–40%) was controlled each experimental day by comparing sensor *I-V* characteristics in the dark in ambient air and dry S.A. and observed to be minor. In the present work, we focused more on understanding the surface phenomena during gas adsorption and proposing an easy method for discriminating gases using different parts of the sensor and *I-V* curves. However, broader experiments with varied humidity would be beneficial for the practical evaluation of prototype sensors. We also indicate that a baseline correction can be performed when the characteristics in humid and dry air are known.

## Electronic supplementary material

Below is the link to the electronic supplementary material.

Supplementary Material 1

## Data Availability

The detailed data that support the findings of our experimental study are available from the corresponding author upon reasonable request.
