# Supplementary material for: Sensing of NO2, NH3, and C3H6O by graphene-Si Schottky diode at chosen voltage biases
Source: Sci Rep. 2025 Mar 21;15:9829. doi: 10.1038/s41598-025-94473-5 (PMC11928667; doi:10.1038/s41598-025-94473-5)
Supplement: Supplementary file 1 — Supplementary Material 1 [file 41598_2025_94473_MOESM1_ESM.pdf]

Supplementary Material

Sensing of NO_2, NH_3, and C_3H_6O by Graphene-Si Schottky Diode at Chosen Voltage Biases

Katarzyna Drozdowska[a,*], Janusz Smulko[a], Adil Rehman[b], Bartłomiej Stonio[c], Aleksandra Krajewska[b], Sergey Rumyantsev[b], Grzegorz Cywiński[b]

[a]*Department of Metrology and Optoelectronics, Faculty of Electronics, Telecommunications, and Informatics, Gdańsk University of Technology, G. Narutowicza 11/12, 80-233, Gdańsk, Poland*

[b]*CENTERA Laboratories, Institute of High Pressure Physics PAS, Warsaw, Poland*

[c]*Centre for Advanced Materials and Technologies CEZAMAT, Warsaw University of Technology, Poleczki 19, 02–822 Warsaw, Poland*

*Corresponding author – katarzyna.drozdowska@pg.edu.pl

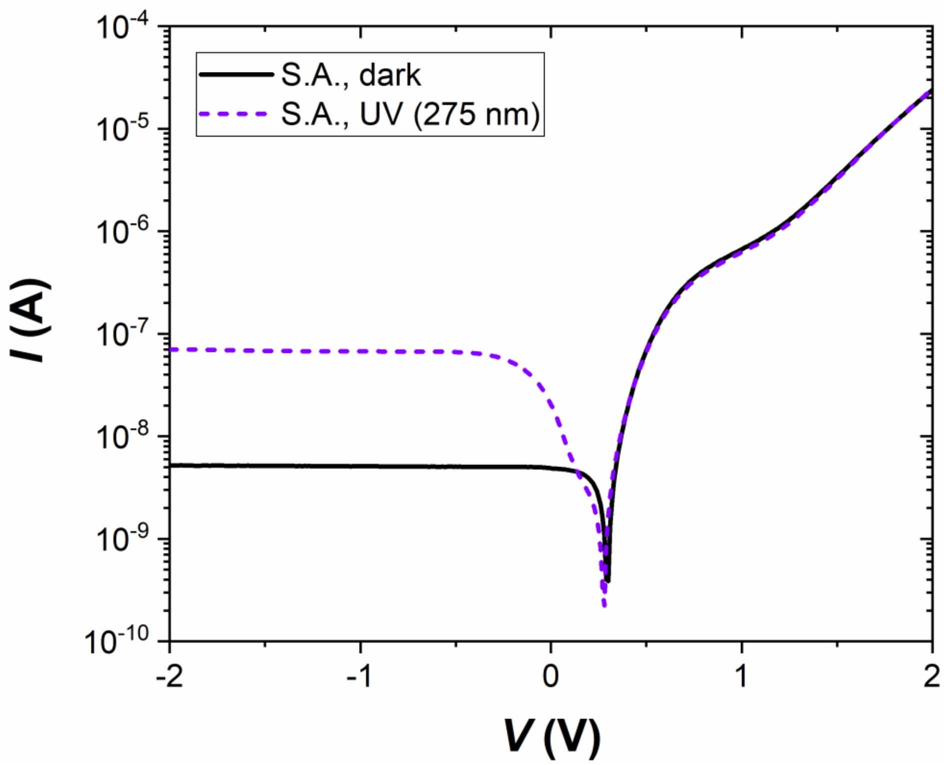

Figure S1 Current-voltage characteristics of the investigated G-Si Schottky diode measured in S.A. in the dark and under UV light (275 nm, 1.59 mW/cm^2) with the shift of ~0.27–0.28 V of the minimum current point, induced by a thermoelectric voltage in the input measurement circuit. The most significant difference between irradiated and dark sensor is in the region of reverse current due to the photovoltaic effect.

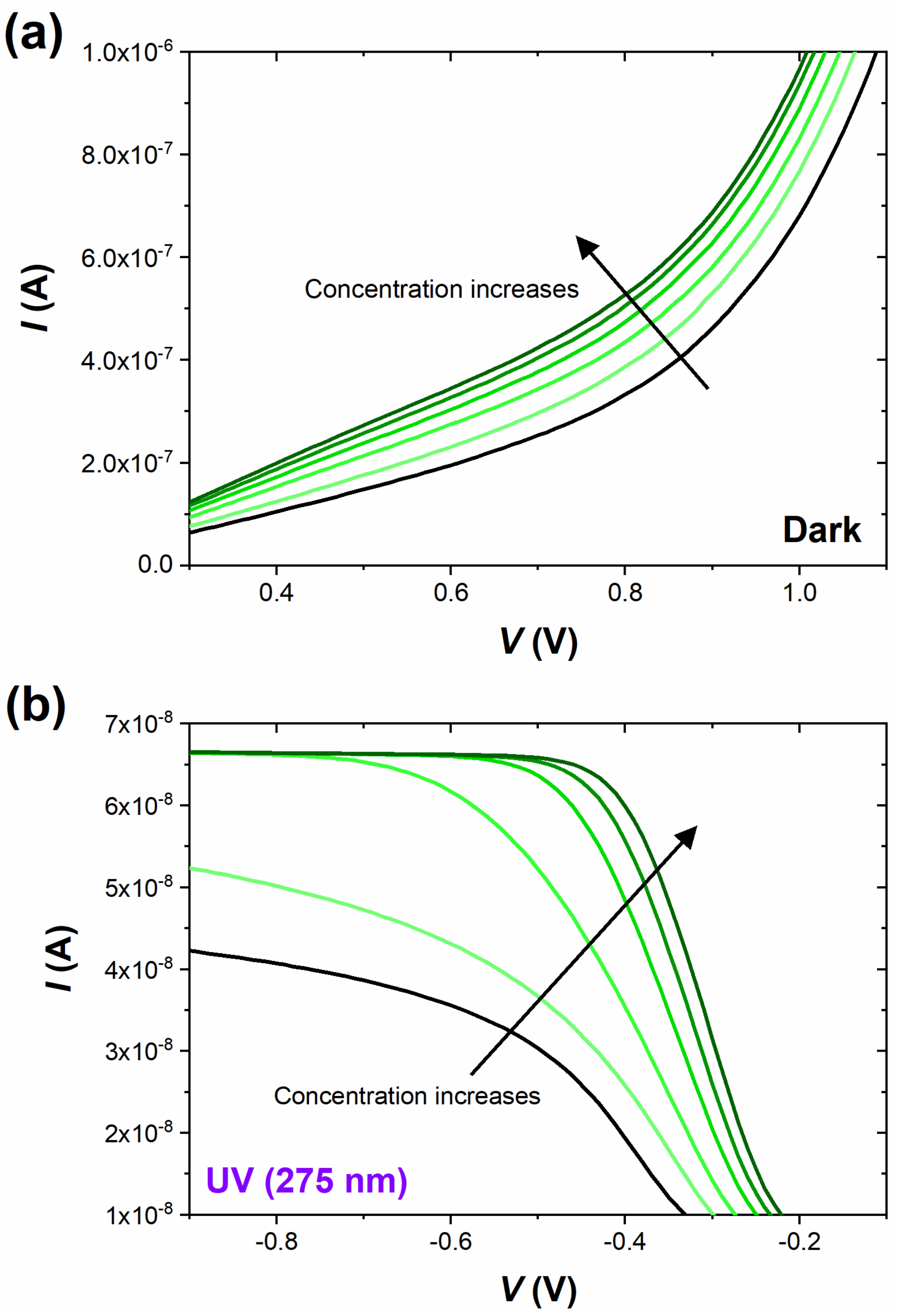

Figure S2 Current-voltage characteristics of the investigated G-Si Schottky diode measured in S.A. and selected concentrations of NO_2 (1–3 ppm) **(a)** in the dark with magnification of the forward bias region ~0.7 V, and **(b)** *I-V* characteristic measured under UV light (275 nm, 1.59 mW/cm^2) with magnified region in the reverse voltage bias with the highest gas responsivity.

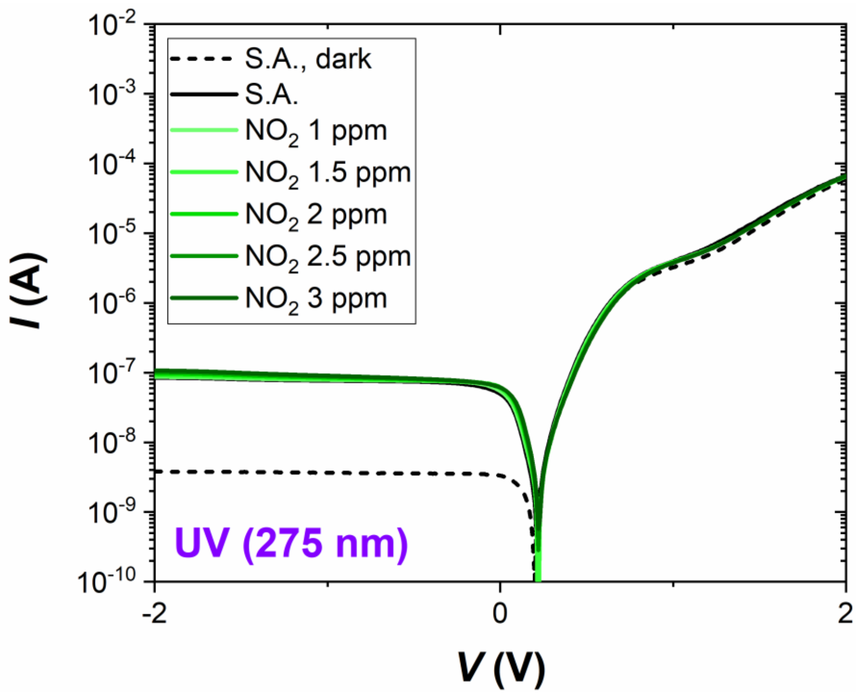

Figure S3 Current-voltage characteristics of the investigated G-Si Schottky diode (second sample) measured in S.A. and selected concentrations of NO_2 (1–3 ppm) under UV light (275 nm, 1.59 mW/cm^2). The black dashed curve is the result for dark conditions to show the effect of UV light in the reverse regime.

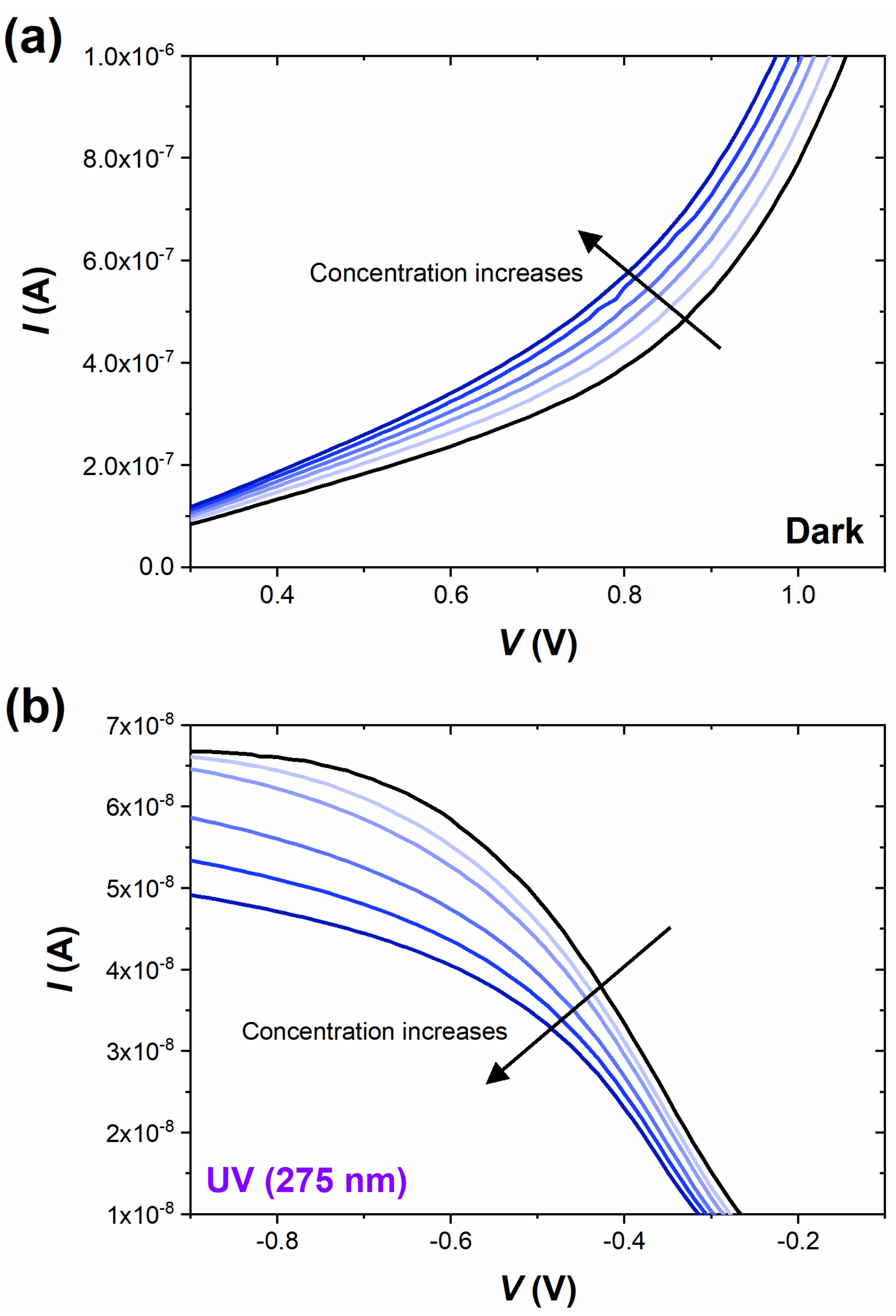

Figure S4 Current-voltage characteristics of the investigated G-Si Schottky diode measured in S.A. and selected concentrations of NH_3 (5–15 ppm) **(a)** in the dark with magnification of the forward bias region ~0.7 V, and **(b)** *I*-*V* characteristic measured under UV light (275 nm, 1.59 mW/cm^2) with magnified region in the reverse voltage bias with the highest gas responsivity.

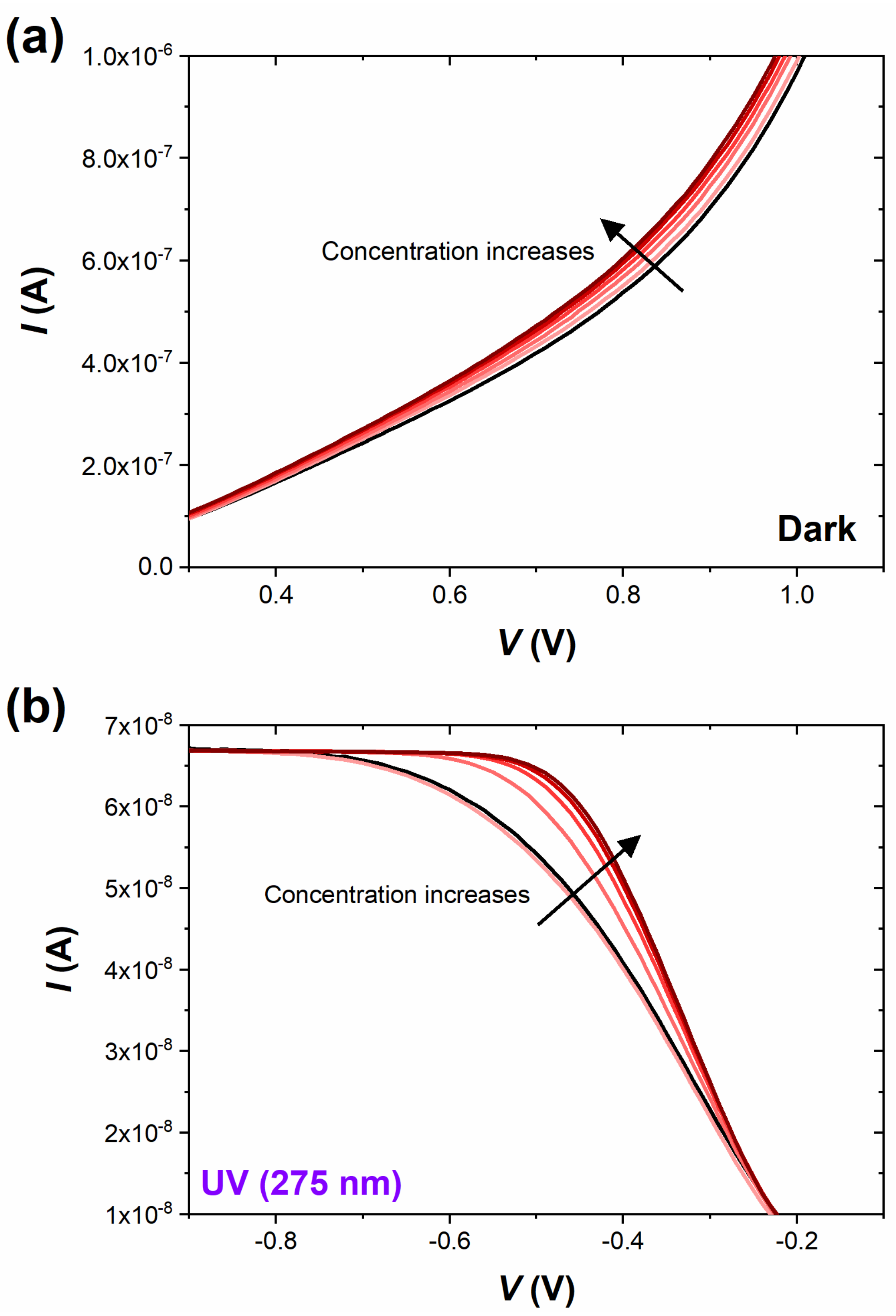

Figure S5 Current-voltage characteristics of the investigated G-Si Schottky diode measured in S.A. and selected concentrations of C_3H_6O (10–30 ppm) **(a)** in the dark with magnification of the forward bias region ~0.7 V, and **(b)** *I*-*V* characteristic measured under UV light (275 nm, 1.59 mW/cm^2) with magnified region in the reverse voltage bias with the highest gas responsivity.

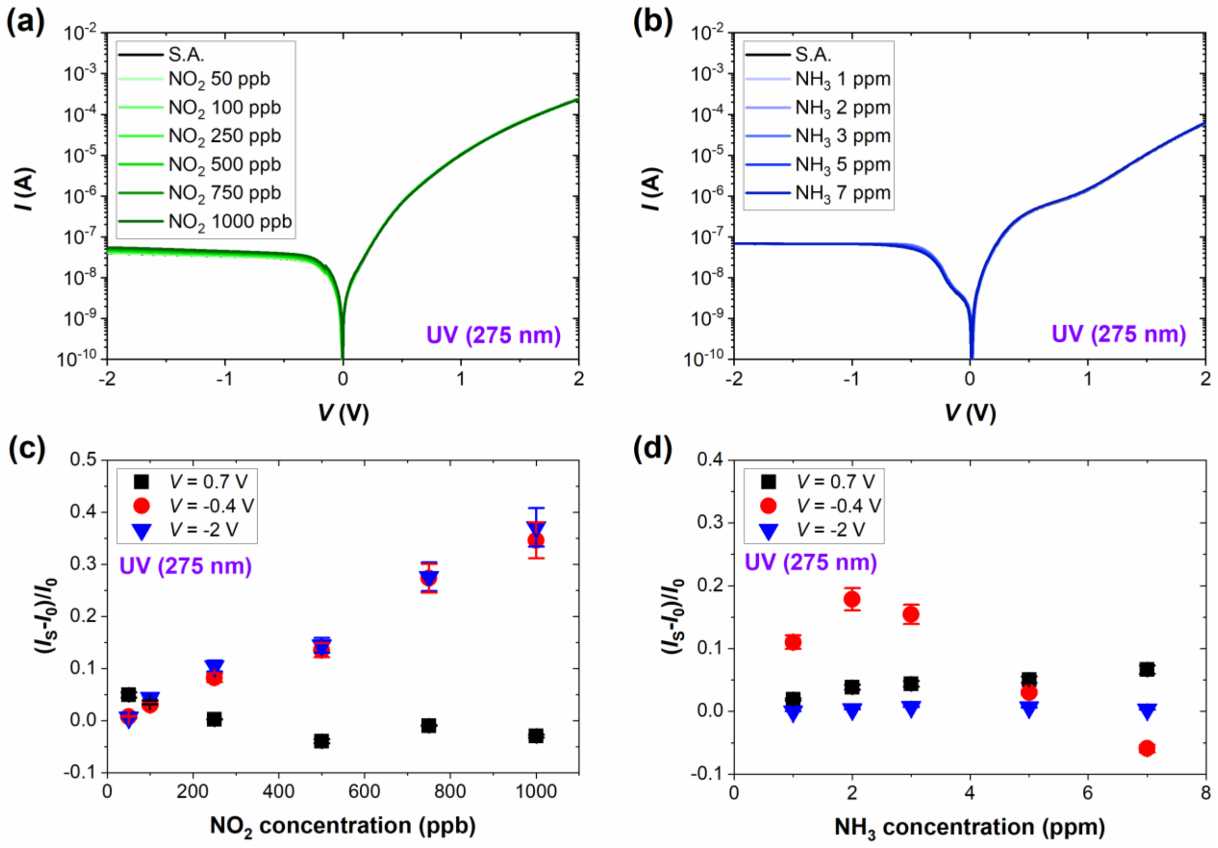

Figure S6 Current-voltage characteristics of the investigated G-Si Schottky diode measured for lower concentrations of **(a)** NO_2 (50–1000 ppb) and **(b)** NH_3 (1–7 ppm) under UV light (275 nm, 1.59 mW/cm^2). The points in **(c)** and **(d)** are derived from the corresponding characteristics at selected voltage bias of 0.7 V, -0.4 V, and -2 V. The error bars represent the accuracy of current measurements.

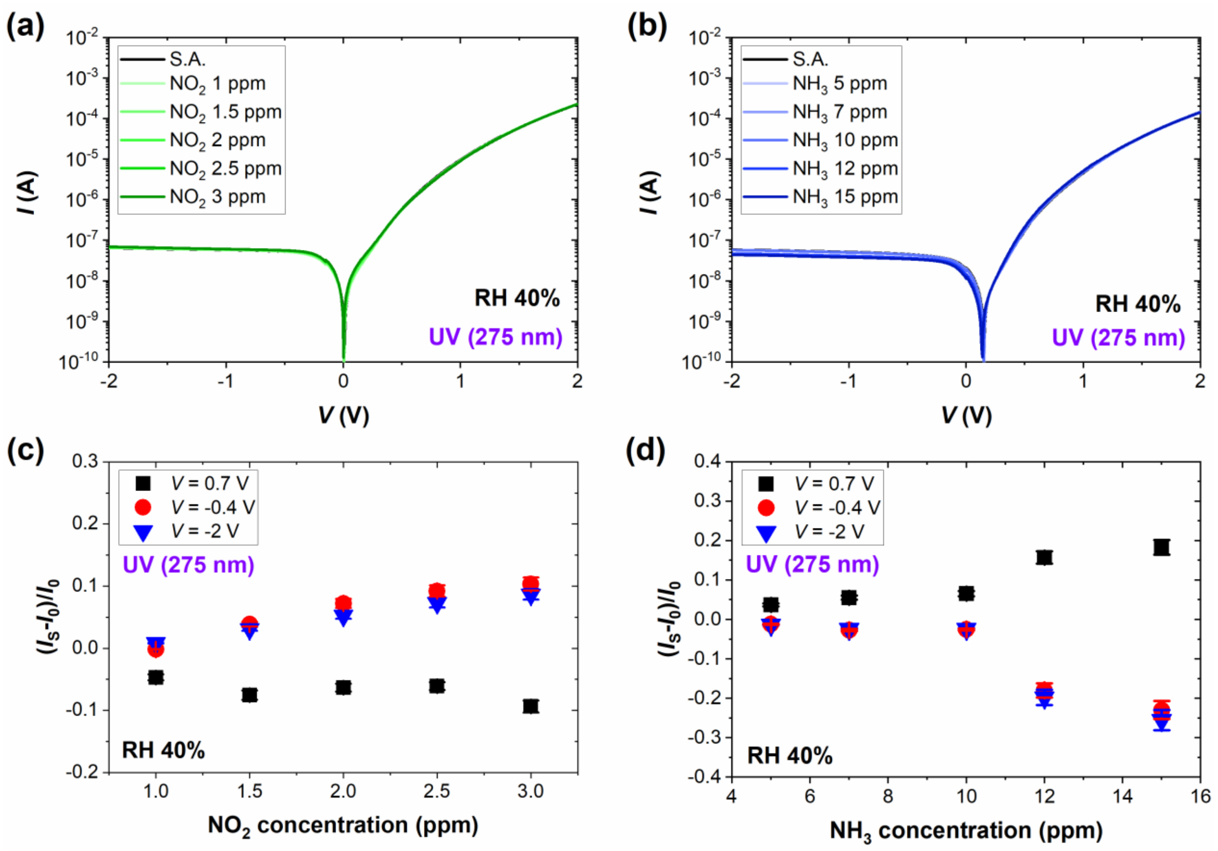

Figure S7 Current-voltage characteristics of the investigated G-Si Schottky diode measured for **(a)** NO_2 (1–7 ppm) and **(b)** NH_3 (5–15 ppm) under UV light (275 nm, 1.59 mW/cm^2) and humid conditions (RH of ~40%). The points in **(c)** and **(d)** are derived from the corresponding characteristics at selected voltage bias of 0.7 V, -0.4 V, and -2 V. The error bars represent the accuracy of current measurements.

(a)

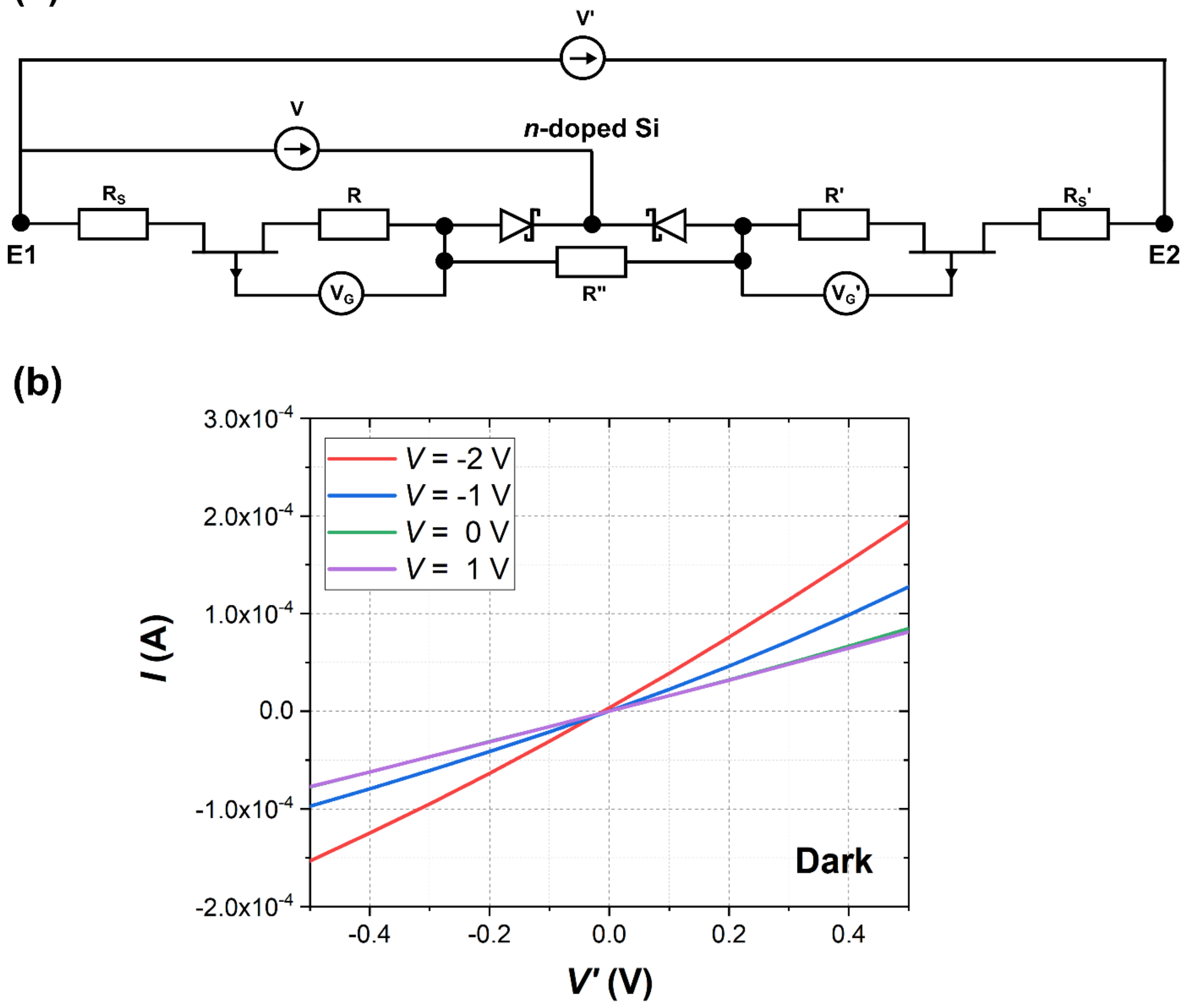

Figure S8 (a) The equivalent circuit proposed for the G-Si sensor for measurements with additional voltage *V*' polarizing the graphene layer deposited between two Ni/Au contacts – E1 and E2. The whole circuit constitutes the reflection of two smaller circuits consisting of sensor resistance R_S or R_S' dominated by resistance of graphene and Ni/Au contact electrode E1 or E2, field-effect transistor part with voltage bias V_G or V_G' induced by dielectric dipoles of the adsorbed gas molecules, and the Schottky diodes representing the junction between graphene and Si. Additional voltage *V*' can be used to measure characteristics of the graphene layer as a function of *V* across the Schottky junction, depicted in **(b)**. Additional resistance *R*" can also appear between two devices with Schottky junctions. The characteristics were measured in the dark and laboratory air for *V*' between -0.5 V and 0.5 V. Larger voltages were not employed so as not to destroy the sample since, at 0.5 V, the current flowing through graphene was already in the hundreds of μA range.

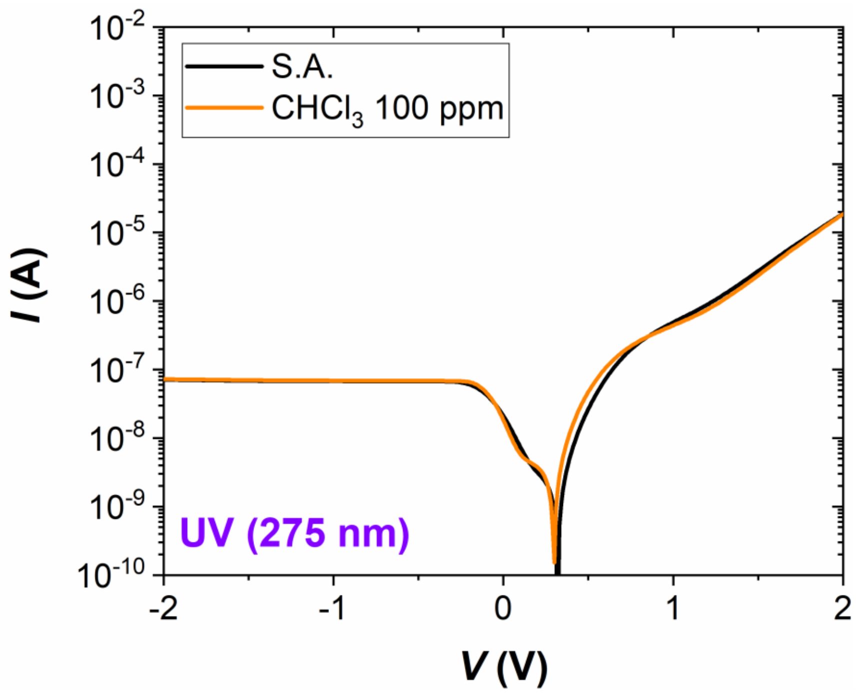

Figure S9 Current-voltage characteristics of the investigated G-Si Schottky diode measured for 100 ppm of chloroform ($CHCl_3$) under UV light (275 nm, 1.59 mW/cm^2). Chloroform induces the changes in current flowing through the G-Si junction in the same direction as oxidizing NO_2. However, the current response is only 2% at -0.4 V compared to 28% for NO_2.
